# Trousseau’s Syrup of Lime in the Treatment of Acute Rheumatism

**Published:** 1867-06

**Authors:** 


### Trousseau's Syrup of Lime in the Treatment of Acute Rheumatism.

We find in the *Boston Medical and Surgical Journal,* for February 28th, the following statement in regard to the use of lime in Rheumatism:

"Having for a year past used what I consider a new remedy for rheumatism, and with better success than from any other remedy, I consider it proper to ask the profession to make a trial of it. It is the syrup of lime, made according to Trousseau's prescription, as found in *Parrish's Pharmacy.* I have used it, according to the severity of the case and the age of the patient, in the dose of ten drops to forty-five drops, and repeated it from two to six hours, as symptoms have seemed to demand. In but one case has any opiate been required from the beginning. Two cases were complicated with Bright's disease, as indicated by the great abundance of albumen and the casts, as seen in the urine. In one of these the albuminuria entirely disappeared, and in the other it has been largely diminished.

"There has been no constipation, but generally looseness of the bowels, after a couple of days' treatment.

"The medicine is best taken in unskimmed milk, in quantity from a tablespoonful to four ounces, according to the size of the dose of syrup."

